# Use of recombinant factor VIIa in uncontrolled gastrointestinal bleeding after hematopoietic stem cell transplantation among patients with thrombocytopenia

**DOI:** 10.12669/pjms.316.8357

**Published:** 2015

**Authors:** Yaqiong Tang, Qian Wu, Xiaojin Wu, Huiying Qiu, Aining Sun, Changgeng Ruan, Depei Wu, Yue Han

**Affiliations:** 1 Yaqiong Tang, Jiangsu Institute of Hematology, Key Laboratory of Thrombosis & Hemostasis of Ministry of Health, The First Affiliated Hospital of Soochow University, Suzhou, China. Collaborative Innovation Center of Hematology, Soochow University, Suzhou, China; 2 Qian Wu, Jiangsu Institute of Hematology, Key Laboratory of Thrombosis & Hemostasis of Ministry of Health, The First Affiliated Hospital of Soochow University, Suzhou, China. Collaborative Innovation Center of Hematology, Soochow University, Suzhou, China; 3 Xiaojin Wu, Jiangsu Institute of Hematology, Key Laboratory of Thrombosis & Hemostasis of Ministry of Health, The First Affiliated Hospital of Soochow University, Suzhou, China. Collaborative Innovation Center of Hematology, Soochow University, Suzhou, China; 4 Huiying Qiu, Jiangsu Institute of Hematology, Key Laboratory of Thrombosis & Hemostasis of Ministry of Health, The First Affiliated Hospital of Soochow University, Suzhou, China. Collaborative Innovation Center of Hematology, Soochow University, Suzhou, China; 5 Aining Sun, Jiangsu Institute of Hematology, Key Laboratory of Thrombosis & Hemostasis of Ministry of Health, The First Affiliated Hospital of Soochow University, Suzhou, China. Collaborative Innovation Center of Hematology, Soochow University, Suzhou, China; 6 Changgeng Ruan, Jiangsu Institute of Hematology, Key Laboratory of Thrombosis & Hemostasis of Ministry of Health, The First Affiliated Hospital of Soochow University, Suzhou, China. Collaborative Innovation Center of Hematology, Soochow University, Suzhou, China; 7 Depei Wu, Jiangsu Institute of Hematology, Key Laboratory of Thrombosis & Hemostasis of Ministry of Health, The First Affiliated Hospital of Soochow University, Suzhou, China. Collaborative Innovation Center of Hematology, Soochow University, Suzhou, China; 8 Yue Han, Jiangsu Institute of Hematology, Key Laboratory of Thrombosis & Hemostasis of Ministry of Health, The First Affiliated Hospital of Soochow University, Suzhou, China. Collaborative Innovation Center of Hematology, Soochow University, Suzhou, China

**Keywords:** Recombinant factor VIIa, gastrointestinal bleeding, hematopoietic stem cell transplantation, thrombocytopenia, hemostatic treatments

## Abstract

**Background and Objective:**

Recombinant-activated factor VII (rVIIa) is a vitamin K-dependent glycoprotein that is an analog of the naturally occurring protease. It has an off-label use to control life-threatening bleeding that is refractory to other measures and was shown to decrease transfusion requirements. Gastrointestinal (GI) bleeding is a severe complication following hematopoietic stem cell transplantation (HSCT) in patients with thrombocytopenia, while hemostatic measures based on antifibrinolytic or transfusion therapy may not always be successful. The present study investigated the treatment with rFVIIa in severe GI bleeding among thrombocytopenia patients undergoing HSCT.

**Methods:**

rFVIIa was given as a single dose of 60μg/kg in patients with GI bleeding following hematopoietic stem cell transplantation (HSCT).

**Results:**

Among all patients enrolled, 12 (75%) of 16 patients obtained a response, of which 5 achieved a complete response and 7 achieved a partial response. The 4 remiaing patients (25%) had no response. Nine patients (56.3%) died in a follow-up of 90 days. No thromboembolic events wereassociated with the drug administration occurred.

**Conclusions:**

Our study showed that rFVIIa may represent an additional therapeutic option in such cases.

## INTRODUCTION

Bleeding complications are common and potentially fatal in patients undergoing hematopoietic stem cell transplantation (HSCT). Gastrointestinal (GI) bleeding, which is observed in 5% - 15% of patients after HSCT,^1^ is one of the most severe bleeding and associated with a high rate of mortality. Severe thrombocytopenia, graft-versus-host disease (GVHD), infections, the anticoagulation therapy, and conditioning regimen contribute to the development of GI bleeding.^2^,^3^ In order to achieve and maintain a favorable outcome of HSCT, successful management of GI bleeding is essential. Unfortunately, the existing therapeutic strategies are often frustrating.

Routine hemostatic treatments, which include platelets transfusion, application of antifibrinolytic agents, and the use of fresh frozen plasma, however, often have limited effects in controlling GI bleeding following HSCT. Furthermore, the limited availability of platelet concentrates and the development of patients’ allo-immunization with consequent platelet refractoriness strongly suggest alternative therapeutic measures.^4^

Recombinant activated factor VII (rFVIIa, NovoSeven) is a systemic prothrombotic agent approved for use in the setting of hemophilia A or B and inhibitors to factor VIII or factor IX,^5^ but now has been increasingly used in the management of off-label indications involving uncontrolled hemorrhage and various surgical scenarios, including excessive intraoperative and postoperative bleeding. We report our preliminary experience in using rFVIIa among hematopoietic malignancy patients suffering from uncontrolled GI bleeding after HSCT.

## METHODS

Patients with hematological malignancies suffering from GI bleeding following HSCT were enrolled in this study. The inclusion criteria were: (1) Severe thrombocytopenia (platelet count < 30 × 10^9^/L); (2) Severe GI bleeding (bleeding score 3-4); (3) No response to traditional hemostatic treatments (no change in type of bleeding or transfusion requirement 72 hours after the administration). Those patients with malignancy relapse or transplant-related thrombosis were excluded. The degree of bleeding was evaluated through a score proposed by Nevo et al.^1^ Score 3 was assigned for hemorrhage which caused rapid decreases in hematocrit level necessitating one or more units of RBC transfusions per day over the expected rate of an individual or failure to obtain a post-transfusion increment. Life-threatening hemorrhage was defined as either massive bleeding causing severe hemodynamic compromise or bleeding into a vital organ resulted in score 4. Severe bleeding included score 3 for 3 or more days (within 7 days) or any score 4. GVHD was diagnosed and scored by the classification scheme, first developed by Klingebiel and Schlegel.^6^

A preparative regimen of modified busulfan and cyclophosphamide was adopted. For patients with matched related donors (MRD), GVHD prophylaxis consisted of cyclosporine A (CSA) and methotrexate (MTX). For those with mismatched related donors (MMRD) and matched unrelated donors (MUD), GVHD prophylaxis should include antithymocyte globulin and mycophenolate mofetil together with CSA and MTX. Platelet transfusions were administered routinely in all bleeding patients. Packed red blood cells were given to achieve a minimal hemoglobin level of 60 g/L. Fresh frozen plasma, cryoprecipitation and prothrombin complex were given to correct coagulopathy. Antifibrinolytic agents (e.g. p-aminomethy benzoic acid, etamcylate) were administrated routinely.

rFVIIa treatment was initiated once the failure of the traditional hemostatic methods was identified. rFVIIa (NovoSeven®, Novo Nordisk A/S, Denmark) was given as a single dose of 60μg/kg intravenously. Platelet infusion was continued during rFVIIa administration to provide a substrate useful for the action of the drug. Criteria for therapeutic efficacy was evaluated as follows:^7^ Complete response (CR), no transfusion requirement, or change from severe to minor type of bleeding); Partial response (PR), reduction of bleeding from severe to moderate; No response (NR), no change in transfusion requirement. Treatment efficacy was evaluated within 72 hours after the treatments. Daily clinical records including blood components infusion and the coagulation index during the study period were collected.

All patients provided written informed consent in accordance with the Declaration of Helsinki, and the study was approved by the ethical committees of our institution.

## RESULTS

From January 2012 to May 2014, 61 patients were complicated with GI bleeding following HSCT, and 16 eligible patients were enrolled in this study. Detailed patient characteristics are listed in Table-I. All patients suffered from severe GI bleeding with a median bleeding score of 4. The median platelet counts in these patients were 16×10^9^/L at the time of bleeding. Five out of 16 patients complicated with platelet transfusion refractoriness. Seven patients suffered from grade IV GVHD and 5 patients suffered from grade III GVHD after HSCT. Anti-GVHD treatment included CSA, tacrolimus, mycophenolate mofetil, methylprednisolone, MTX, cyclophosphamide, anti-CD25 mAb, etc. Hemorrhagic cystitis occurred in patient No. 3, and routine therapy of forced hyperhydration and continuous bladder irrigation were performed. Meanwhile, 4 patients suffered from active pneumonia and received antibiotic therapy routinely; three patients complicated with mucositis in the process of pretreatment.

**Table-I:** The clinical and laboratory characteristics of patients.

Case	Sex/Age	Primary disease	HSCT	PLTS (x109/L)	Complication	Platelet TR	Bleeding score	Response	Outcome (days after rVIIa)
1	F/42	AML	MRD +45d	29	IV GVHD	No	4	PR	Death (10)
2	M/18	AML	MMRD +143d	28	III GVHD	No	4	PR	Survival (90)
3	M/15	ALL	MUD +50d	9	IV GVHD; HC	Yes	3	CR	Death (16)
4	M/19	AML	MMRD +189d	17	III GVHD; pneumonia	Yes	4	NR	Survival (90)
5	M/13	ALL	MMRD +31d	30	IV GVHD	No	3	NR	Death (26)
6	F/51	lymphoma	MRD +265d	15	IV GVHD	No	4	NR	Death (24)
7	M/38	AML	MMRD +101d	25	III GVHD	No	4	PR	Death (49)
8	M/50	ALL	MMRD +66d	9	IV GVHD	Yes	4	NR	Death (2)
9	M/18	ALL	MUD +228d	10	IV GVHD; pneumonia	Yes	4	CR	Death (18)
10	M/34	ALL	MRD +116d	28	IV GVHD; pneumonia	No	3	PR	Death (29)
11	M/40	CML	MMRD +111d	3	III GVHD	No	3	CR	Survival (90)
12	M/22	ALL	MUD +163d	26	pneumonia	No	4	PR	Survival (90)
13	M/27	ALL	MMRD +209d	5	III GVHD pneumonia	No	4	PR	Death (32)
14	F/33	CML	MUD -3d	30	Mucositis	No	4	CR	Survival (90)
15	F/48	AML	MMRD -3d	11	Mucositis	Yes	4	CR	Survival (90)
16	M/31	AML	MMRD -1d	15	Mucositis	No	3	PR	Survival (90)

Twelve of 16 patients in our case series (75%) obtained a response to rFVIIa therapy, of which 5 patients achieved a CR and 7 patients achieved a PR. Four other patients (25%) did not response. Nine patients died in a follow-up of 90 days with a mortality of 56.3%, of which five patients died of organ failure, two patients died of uncontrolled GVHD and two died of uncontrolled bleeding. Furthermore, no thromboembolic complications associated with rFVIIa treatment was observed.

**Fig.1:**
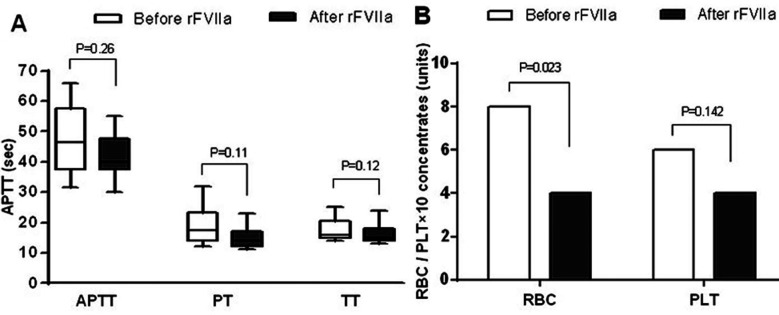
The coagulation index and blood components infusion before and after rFVIIa administration (within 72h). (A) APTT, PT and TT values did not changed significantly after rFVIIa administration (P>0.05). (B) The packed red cells infused decreased after rFVIIa treatment (P<0.05), the volume of fresh platelet was comparable (P>0.05).

Activated partial thromboplastin time (APTT), prothrombin time (PT) and thrombin time (TT) did not change significantly after rFVIIa treatment (P>0.05) (Figure 1-A). The packed red cells infusion decreased after rFVIIa administration (within 72 hours) (P<0.05), whereas the volume of fresh platelet and fresh-frozen plasma infusion was comparable before and after rFVIIa treatment (within 72 hours) (Fig.1-B).

## DISCUSSION

GI bleeding is an important cause of morbidity and mortality in patients with hematologic malignancies following HSCT, resulted in a mortality of 34-40% in bleeding patients.^8^ Endothelial cells injury and profound thrombocytopenia are the major components involved in GI bleeding.^9^ GVHD strongly increases the risk of bleeding by direct local effects. In tissues affected by acute and chronic GVHD, hyperperfusion and proliferation of blood vessels may lead to increased epithelial fragility and subsequent destruction, further increasing the risk of hemorrhagic complications.^10^ The probability of hemostatic treatment failure is high if the patient is bleeding from severe GVHD.^8^ In our case series, four patients with no response to rFVIIa therapy were complicated with III-IV GVHD after HSCT, suggesting that control of GI bleeding with GVHD is more difficult, and management of GVHD and prevention further damage to tissue and vessel may do help in such case.

Thrombocytopenia is closely related to bleeding episodes in patients following HSCT.^2^,^3^ If platelet refractoriness is associated with GI bleeding, routine hemostatic interventions may not help.^11^ Most patients in our case series remained dependent on platelet transfusion, five patients became platelet transfusion refractoriness, which would contribute to the failure of the hemostatic therapy.

Because of the unique mechanism of action of rFVIIa that is dependent on forming a complex with tissue factor (TF) and generating sufficient thrombin at the site of injury, the drug is increasingly used in “off-label” situations to enhance hemostasis in non-hemophilic patients who experience bleeding episodes unresponsive to conventional therapies.^12^-^15^ Recently published data on rFVIIa off-label use in 45 non-hemophiliac patients showed that overall transfusion requirements significantly decrease after the infusion of rFVIIa, 19 patients (42.2%) died and thrombosis was documented in 3 patients (6.7%).^16^ In models of thrombocytopenia, rFVIIa can also facilitate hemostasis by increasing the initial thrombin generation.^17^ Until now, the use of this agent in patients with hematological malignancies suffering from GI bleeding following HSCT has been mostly described in anecdote case reports.^7^,^11^,^15^ The only randomized trial available in the literature was published by Pihusch *et al*.^18^ One hundred patients with moderate or severe bleeding (including 52 gastrointestinal) following HSCT received seven doses of rFVIIa, an improvement in the control of bleeding for the rFVIIa 80μg/kg group versus the standard hemostatic treatment group was observed, while there were no differences in transfusion requirements across dose groups (40, 80 or 160μg/kg). In addition, Franchini M *et al* reviewed 113 HSCT patients suffering from hemorrhage, GI bleeding was the leading cause (46.9%), 69 out of 113 patients (61.1%) had a cessation or significant reduction of bleeding after rFVIIa treatment.^19^

However, the real effectiveness of rFVIIa in these bleeding situations could be overestimated, those cases with a positive outcome being preferentially reported. Eller P and colleagues presented an ineffective use of rFVIIa in a case of HSCT-related GI bleeding. Despite more than 10 doses of 90-120μg/kg, recurrent severe bleeding progressed to refractory shock, multiorgan failure and death.^12^ Another case reported by Millar *et al* showed no response to rFVIIa in a 3-year old patient complicated with sever GI bleeding following HSCT.^13^ These cases suggested that rFVIIa is not a panacea, especially for life-threatening bleeding following HSCT, management of the underlying condition will do help in such case.

Until now, there is no recommendation about the optimum dosage of rFVIIa for the management of bleeds in patients with thrombocytopenia related to hematologic malignancies following HSCT. Due to financial limitation of the majority patients in China, repeated application of rFVIIa would be difficult. Our patients received rFVIIa with one single dose of 60.0μg/kg, less than 96.6μg/kg reported by M Franchini *et al*, while the response rate was comparable.

Thromboembolic complication is the major concern of rFVIIa administration. O’Connell KA *et al*. analysed 431 adverse events reports of rFVIIa, of which 168 reports described 185 thromboembolic events.^5^ About 52% events occurred in the first 24 hours after the last dose. In 36 (72%) of 50 reported deaths, the probable cause of death was the thromboembolic event. Levi M analyzed data from 35 randomized clinical trials and concluded that treatment with high doses of rFVIIa significantly increased the risk of arterial but not venous thromboembolic events, especially among the elderly.^20^ However, no thromboembolic complications occurred in our case series, which was possibly ascribed to the lower dosage.

We conclude that rFVIIa should be considered as an alternative mode of therapy for life-threatening GI bleeding in thrombocytopenia patients following HSCT. Further large randomized trials should focus on evaluating and defining the most appropriate usage and dosage of rFVIIa in bleeding HSCT patients.
